# Irving S. Wright Award of Distinction Lecture, Vincent Cristafalo Award Lecture, and Terrie Fox Wetle Award Lecture

**DOI:** 10.1093/geroni/igab046.1488

**Published:** 2021-12-17

**Authors:** Nir Barzilai, Terrie Fox Wetle

## Abstract

The Irving S. Wright Award of Distinction Lecture will feature an address by the 2021 recipient Malene Hansen, PhD of the Buck Institue for Research on Aging. The Vincent Cristofalo Rising Star Award in Aging Research lecture will feature an address by the 2021 recipient, Morgan Levine, PhD, of Yale University. The Terrie Fox Wetle Award lecture will feature an address by the 2020 recipient, Kali Thomas, PhD, FGSA of Brown University and an address by the 2021 recpient, Andrea Gilmore-Bykovskyi of the University of Wisconsin, Madison. These awards are given by the American Federation for Aging Research, Inc.

